# Draft Genome Sequences of Three Cellulolytic *Bacillus licheniformis* Strains Isolated from Imperial Geyser, Amphitheater Springs, and Whiterock Springs inside Yellowstone National Park

**DOI:** 10.1128/genomeA.00065-17

**Published:** 2017-03-30

**Authors:** Joshua A. O' Hair, Hui Li, Santosh Thapa, Matthew Scholz, Suping Zhou

**Affiliations:** a Department of Agricultural and Environmental Sciences, Tennessee State University, Nashville, Tennessee, USA; b Vanderbilt Technologies for Advanced Genetics (VANTAGE), Vanderbilt University Medical Center, Nashville, Tennessee, USA

## Abstract

Novel cellulolytic microorganisms are becoming more important for rapidly growing biofuel industries. This paper reports the draft genome sequences of *Bacillus licheniformis* strains YNP2-TSU, YNP3-TSU, and YNP5-TSU. These cellulolytic isolates were collected from several hydrothermal features inside Yellowstone National Park.

## GENOME ANNOUNCEMENT

As the first national park, Yellowstone has had a long-documented history of preservation, making it an ideal location to study thermophilic specimens in their natural state (1). Three heat-tolerant cellulolytic *Bacillus licheniformis* isolates were identified through genome sequencing. The first, YNP2-TSU, was removed from Amphitheater Springs (latitude [lat] 44.8016, longitude [long] -110.7288), a hydrothermal feature southeast of the Solfatara Creek trailhead. Isolate YNP3-TSU was removed from Imperial Geyser runoff (lat 44.5316, long -110.8760) approximately 3 mi east along Fairy Falls trail, and the third isolate, YNP5-TSU, was collected near the confluence of two small creeks flowing through Whiterock Springs (lat 44.7803, long -110.6981). From each sampling site, 50 ml of water was vacuum-filtrated through 0.22-µm-pore filters. Filters were then transferred to nutrient agar, and areas with substantial growth were restreaked to produce individual colonies (2). The bacterial strains reported here tested positive for extracellular endoglucanase activity on 10% carboxymethylcellulose (CMC) under the Congo Red assay (3). After positive cellulase testing, whole-genomic DNA was extracted using GenElute Sigma genomic DNA kit for Gram-positive strains (Sigma, CA).

Libraries were prepared with TruSeq DNA Nano sample kits using indexed adaptors (Illumina). Pooled libraries were subjected to 150-bp paired-end sequencing, according to the manufacturer’s protocol (Illumina HiSeq 3000). The bcl2fastq2 Conversion Software (Illumina) was used to generate demultiplexed Fastq files. This work was performed at the Vanderbilt Technologies for Advanced Genomics (VANTAGE) at Vanderbilt University (Nashville, TN). Raw reads were then trimmed to remove bases of average *Q* ≤3 using the BWA method (4). *De novo* assembly was performed using SPAdes version 3.7.1 (5), with default parameters and the -careful flag.

The draft genome of YNP2-TSU was assembled into 96 contigs, with a total genome size of 4,765,942 bp (*N*_50_, 521,225 bp) and a G+C content of 45.0%. Automated annotation was performed by the NCBI Prokaryotic Genome Annotation Pipeline (PGAP, version 3.3) and yielded 4,822 coding sequences (CDSs), 88 tRNAs, and 16 rRNAs. The draft genome of YNP3-TSU was assembled into 53 contigs, with a total genome size of 4,518,376 bp (*N*_50_, 706,022 bp) and a G+C content of 45.3%. Through the NCBI PGAP (version 3.3) 4,822 CDSs, 88 tRNAs, and 16 rRNAs were predicted. The third genome, YNP5-TSU, was assembled into 65 contigs, with a genome size of 4,536,725 bp (*N*_50_, 747,706 bp) and G+C content of 45.5%. Annotation also predicted 4,523 CDSs, 83 tRNAs, and 16 rRNAs. With respect to all three genomes, several endoglucanase, beta-cellobiosidase, exo-1,4-beta-glucosidase, and beta-xylosidase genes were predicted. Enzymes were categorized into predicted glycoside hydrolase (GH) families 1, 3, 9, 43, and 48 (UniProt) (6). GH9, GH1, and GH3 also fell into carbohydrate-binding module families X2, 3, and 6, suggesting a cellulosome hierarchy in all three strains (7). Future work to purify and test enzymatic functions will be of great importance in evolving biofuel production.

### Accession number(s).

The whole-genome shotgun projects have been deposited at DDBJ/EMBL/GenBank under the accession numbers MEDB00000000 (YNP2-TSU), MEDC00000000 (YNP3-TSU), and MEDD00000000 (YNP5-TSU) as the first versions.
